# Chronic Kidney Disease: The Silent Epidemy

**DOI:** 10.3390/jcm8111795

**Published:** 2019-10-26

**Authors:** Antonio Bellasi, Luca Di Lullo, Biagio Di Iorio

**Affiliations:** 1 Department of Research, Innovation, Brand Reputation, Ospedale di Bergamo, ASST Papa Giovanni XXIII, 24127 Bergamo, Italy; 2 Department of Nephrology and Dialysis, “L. Parodi–Delfino” Hospital, 00034 Colleferro, Italy; dilulloluca69@gmail.com; 3 Department of Medicine, Nephrology Unit, AORN “Antonio Cardarelli”, 80131 Neaples, Italy; br.diiorio@gmail.com

Numerous observations suggest that chronic kidney disease (CKD) is an epidemic condition [1]. Although its prevalence varies according to geographical region, it has been reported that about 8–10% of the population show some degree of renal function impairment [1]. It is worth noting that CKD not only portends poor prognosis but it has also been demonstrated that CKD patients are among the most complex subjects (complexity being defined as number of comorbidities, presence of mental illness, number of types of physicians involved in each patient’s care, number of physicians involved in each patient’s care, number of prescribed medications, number of emergency department visits, rate of death, rate of hospitalization, and rate of placement in a long-term care facility) to be managed, and consumes a large proportion of health care resources [2].

Contrary to diabetes or other metabolic diseases as prevalent as CKD, renal function impairment is often asymptomatic or pauci-symptomatic until very late stages of the disease. However, in the course of the disease many metabolic abnormalities may develop and aggravate patients’ well-being and prognosis [3]. Needless to say, great effort is being devoted to characterizing clinical symptoms, developing diagnostics, as well as treatments, to prevent CKD occurrence or progression toward end stage renal disease (ESRD). 

The aim of this special issue is to collect data and share ideas on different aspects of these topics. The 25 papers published (Table 1) and the 26 (highest citations of 9) citations document will be of interest to investigators and readers interested in the complexity of CKD. Four articles [4,5,6,7] investigated the usefulness of various biomarkers to assess renal function or complications associated with CKD in adult, as well as pediatric, patients. Indeed, current strategies to assess renal function rely on serum creatinine levels a marker highly influenced by factors such as age, gender, race, comorbid conditions and/or use of concomitant medications. These articles highlight the need for a more accurate approach to assess the presence and severity of renal impairment [8]. Similarly, nephrologists’ lack of an accurate prognostic score system that integrates serological and histopathological pieces of information is documented in two other studies [9,10] published within this special issue.

Four other articles [11,12,13,14] report on the complicated interplay between CKD and other comorbid conditions. Although prevalence varies according to the region of the world, diabetes and Human Immunodeficiency Virus (HIV) infection are among the most common factors associated with renal dysfunction [12,13,14]. All comorbid conditions need to be taken into account to individualize and improve patient care. However, these articles call for more and dedicated studies to address how to appropriately manage highly comorbid CKD patients, especially in consideration of the fact that these subjects are often excluded by randomized clinical trials (RCT).

Other articles investigate the prevalence and impact of common metabolic abnormalities that frequently complicate the course of CKD in children or adults [15,16,17]. CKD should be perceived as a multifaced metabolic disease that accelerates aging. Anemia, hypoglycemia and phosphate metabolism abnormalities are only some examples of factors that have been associated with Cardio-Vascular (CV) senescence and/or morbity and mortality [15,16,17]. Indeed, as renal function declines, several toxins may accumulate and exert detrimental effects on various metabolic pathways including promoting inflammation and renal damage progression [18,19,20]. In these perspectives, balanced nutrition may modulate the intestinal microbiota [21,22], reduce some toxin productio, and accumulation, and hold promise to impact renal and overall survival of CKD patients [21,22].

Several other therapies have been proposed to tackle the abysmal risk of renal, cardiovascular and all-cause events at which CKD patients are exposed. Overall, eight out of 25 articles (32%) address the impact of different treatment strategies on various outcomes [23,24,25,26,27,28,29]. However, none of these reports on results of an RCT. Although this may be expected for a special issue of a journal of internal medicine, it also reflects the paucity of RCTs in the nephrology arena and the desperate call for more and ad hoc studies to investigate the impact and potential interaction of available drugs on renal function and outcomes of CKD patients.

In conclusion, the articles published in this issue reflect the complexity of CKD and the limitations of available tools to detect renal dysfunction and metabolic abnormalities associated with CKD, as well as the paucity of data to properly treat these patients. Nevertheless, they expand current understanding in some areas of nephrology and generate hypothesis to be tested in future studies.

**Table 1:** List of all published articles with the special issue “Clinical Symptoms, Diagnostics and Treatments of Chronic Kidney Diseases (CKD)” between June 2018 and June 2019.

*N*	Year	Authors	Title	Keywords
1	2018 [4]	Maciejczyk, Mateusz; Szulimowska, Julita; Skutnik, Anna; Taranta-Janusz, Katarzyna; Wasilewska, Anna; Wiśniewska, Natalia; Zalewska, Anna	Salivary Biomarkers of Oxidative Stress in Children with Chronic Kidney Disease	chronic kidney disease; salivary biomarkers; oxidative stress; oxidative damage
2	2018 [21]	Marzocco, Stefania; Fazeli, Gholamreza; Di Micco, Lucia; Autore, Giuseppina; Adesso, Simona; Dal Piaz, Fabrizio; Heidland, August; Di Iorio, Biagio	Supplementation of Short-Chain Fatty Acid, Sodium Propionate, in Patients on Maintenance Hemodialysis: Beneficial Effects on Inflammatory Parameters and Gut-Derived Uremic Toxins, A Pilot Study (PLAN Study)	propionic acid; chronic kidney disease; hemodialysis; gut microbiome; systemic micro-inflammation oxidative stress; indoxyl sulfate; p-cresyl sulfate
3	2018 [18]	Adesso, Simona; Paterniti, Irene; Cuzzocrea, Salvatore; Fujioka, Masaki; Autore, Giuseppina; Magnus, Tim; Pinto, Aldo; Marzocco, Stefania	AST-120 Reduces Neuroinflammation Induced by Indoxyl Sulfate in Glial Cells	indoxyl sulfate; chronic kidney disease; neuroinflammation; glial cells; AST-120
4	2018 [19]	Chaves, Maria; Mendes, Matheus; Schwermann, Maximilian; Queiroz, Raquel; Coelho, Regina; Salmito, Francisco; Meneses, Gdayllon; Martins, Alice; Moreira, Ana; Libório, Alexandre	Angiopoietin-2: A Potential Mediator of the Glycocalyx Injury in Adult Nephrotic Patients	Agiopoietin-2; mediation analysis; nephrotic syndrome
5	2018 [23]	Yang, Chen-Ta; Kor, Chew-Teng; Hsieh, Yao-Peng	Long-Term Effects of Spironolactone on Kidney Function and Hyperkalemia-Associated Hospitalization in Patients with Chronic Kidney Disease	chronic kidney disease (CKD); end-stage renal disease (ESRD); major adverse cardiovascular events (MACE); mortality; spironolactone
6	2018 [5]	Jhee, Jong; Hwang, Seun; Song, Joon; Lee, Seoung	Upper Normal Serum Creatinine Concentrations as a Predictor for Chronic Kidney Disease: Analysis of 14 Years’ Korean Genome and Epidemiology Study (KoGES)	serum creatinine; estimated glomerular filtration rate; chronic kidney disease; proteinuria
7	2018 [11]	Lee, Wen-Chin; Lee, Yueh-Ting; Li, Lung-Chih; Ng, Hwee-Yeong; Kuo, Wei-Hung; Lin, Pei-Ting; Liao, Ying-Chun; Chiou, Terry; Lee, Chien-Te	The Number of Comorbidities Predicts Renal Outcomes in Patients with Stage 3–5 Chronic Kidney Disease	chronic kidney disease; multimorbidity; renal outcomes
8	2018 [9]	Provenzano, Michele; Minutolo, Roberto; Chiodini, Paolo; Bellizzi, Vincenzo; Nappi, Felice; Russo, Domenico; Borrelli, Silvio; Garofalo, Carlo; Iodice, Carmela; De Stefano, Toni; Conte, Giuseppe; Heerspink, Hiddo; De Nicola, Luca	Competing-Risk Analysis of Death and End Stage Kidney Disease by Hyperkalaemia Status in Non-Dialysis Chronic Kidney Disease Patients Receiving Stable Nephrology Care	CKD; ESKD; death; anti-RAS; hyperkalaemia; competing risk
9	2018 [6]	Hoi, Shotaro; Takata, Tomoaki; Sugihara, Takaaki; Ida, Ayami; Ogawa, Masaya; Mae, Yukari; Fukuda, Satoko; Munemura, Chishio; Isomoto, Hajime	Predictive Value of Cortical Thickness Measured by Ultrasonography for Renal Impairment: A Longitudinal Study in Chronic Kidney Disease	ultrasonography; kidney size; cortex; CKD risk factors; kidney function
10	2018 [12]	Hsu, Po-Ke; Kor, Chew-Teng; Hsieh, Yao-Peng	Effect of New-Onset Diabetes Mellitus on Renal Outcomes and Mortality in Patients with Chronic Kidney Disease	chronic kidney disease (CKD); end-stage renal disease (ESRD); incident diabetes; mortality; new-onset diabetes mellitus (DM)
11	2019 [15]	Lee, Keum; Park, Eujin; Choi, Hyun; Kang, Hee; Ha, Il-Soo; Cheong, Hae; Park, Young; Cho, Heeyeon; Han, Kyoung; Kim, Seong; Cho, Min; Lee, Joo; Shin, Jae	Anemia and Iron Deficiency in Children with Chronic Kidney Disease (CKD): Data from the Know-Ped CKD Study	anemia; iron deficiency; chronic kidney disease
12	2019 [24]	Tesfaye, Wubshet; Peterson, Gregory; Castelino, Ronald; McKercher, Charlotte; Jose, Matthew; Zaidi, Syed; Wimmer, Barbara	Medication-Related Factors and Hospital Readmission in Older Adults with Chronic Kidney Disease	chronic kidney disease; medication appropriateness index; medication regimen complexity index; the elderly
13	2019 [17]	Hsiao, Ching-Chung; Tu, Hui-Tzu; Lin, Chi-Hung; Chen, Kuan-Hsing; Yeh, Yung-Hsin; See, Lai-Chu	Temporal Trends of Severe Hypoglycemia and Subsequent Mortality in Patients with Advanced Diabetic Kidney Diseases Transitioning to Dialysis	advanced diabetic kidney disease; severe hypoglycemia; dialysis
14	2019 [7]	Watson, Drew; Yang, Joshua; Sarwal, Reuben; Sigdel, Tara; Liberto, Juliane; Damm, Izabella; Louie, Victoria; Sigdel, Shristi; Livingstone, Devon; Soh, Katherine; Chakraborty, Arjun; Liang, Michael; Lin, Pei-Chen; Sarwal, Minnie	A Novel Multi-Biomarker Assay for Non-Invasive Quantitative Monitoring of Kidney Injury	KIT Assay; chronic kidney disease; biomarker; non-invasive; urine; eGFR; cfDNA
15	2019 [25]	Kim, Chan; Oh, Hyung; Kim, Yon; Kim, Yong-Lim; Chang, Jae; Ryu, Dong-Ryeol	The Effect of Aspirin on Preventing Vascular Access Dysfunction in Incident Hemodialysis Patients: A Prospective Cohort Study in Korean Clinical Research Centers for End-Stage Renal Disease (CRC for ESRD)	aspirin; vascular access failure; incident hemodialysis
16	2019 [26]	Di Micco, Lucia; Di Lullo, Luca; Bellasi, Antonio; Di Iorio, Biagio	Very Low Protein Diet for Patients with Chronic Kidney Disease: Recent Insights	chronic kidney disease; nutritional therapy; urea; phosphorus; metabolic acidosis; vascular calcification; proteinuria; gut; microbioma; cardiovascular risk; very low protein diet
17	2019 [10]	Tsai, Shang-Feng; Wu, Ming-Ju; Wen, Mei-Chin; Chen, Cheng-Hsu	Serologic and Histologic Predictors of Long-Term Renal Outcome in Biopsy-Confirmed IgA Nephropathy (Haas Classification): An Observational Study	IgA nephropathy; patient outcome; renal outcome
18	2019 [27]	Mühlig, Anne; Lee, Jun; Kemper, Markus; Kronbichler, Andreas; Yang, Jae; Lee, Jiwon; Shin, Jae; Oh, Jun	Levamisole in Children with Idiopathic Nephrotic Syndrome: Clinical Efficacy and Pathophysiological Aspects	levamisole; nephrotic syndrome; podocyte; steroid-dependent nephrotic syndrome
19	2019 [16]	Bellasi, Antonio; Di Micco, Lucia; Russo, Domenico; De Simone, Emanuele; Di Iorio, Mattia; Vigilante, Raffaella; Di Lullo, Luca; Di Iorio, Biagio	Fractional Excretion of Phosphate (FeP) Is Associated with End-Stage Renal Disease Patients with CKD 3b and 5	phosphate; phosphate balance; CKD–MBD; outcome; fractional excretion of phosphate; FeP
20	2019 [20]	Mohammed, Chrysan; Xie, Yanmei; Brewster, Pamela; Ghosh, Subhanwita; Dube, Prabhatchandra; Sarsour, Tiana; Kleinhenz, Andrew; Crawford, Erin; Malhotra, Deepak; James, Richard; Kalra, Philip; Haller, Steven; Kennedy, David	Circulating Lactonase Activity but Not Protein Level of PON-1 Predicts Adverse Outcomes in Subjects with Chronic Kidney Disease	paraoxonase; lactonase activity; chronic kidney disease; clinical outcomes
21	2019 [28]	Russo, Domenico; Tripepi, Rocco; Malberti, Fabio; Di Iorio, Biagio; Scognamiglio, Bernadette; Di Lullo, Luca; Paduano, Immacolata; Tripepi, Giovanni; Panuccio, Vincenzo	Etelcalcetide in Patients on Hemodialysis with Severe Secondary Hyperparathyroidism. Multicenter Study in “Real Life”	secondary hyperparathyroidism; cinacalcet; etelcalcetide; hypocalcemia; gastrointestinal side effects
22	2019 [29]	Lee, Yookyung; Kwon, SuYeon; Moon, Jong; Han, Kyungdo; Paik, Nam-Jong; Kim, Won-Seok	The Effect of Health-Related Behaviors on Disease Progression and Mortality in Early Stages of Chronic Kidney Disease: A Korean Nationwide Population-Based Study	chronic kidney disease; disease progression; end stage renal disease; mortality; health-related behaviors; physical activity; smoking; alcohol
23	2019 [13]	Alfano, Gaetano; Cappelli, Gianni; Fontana, Francesco; Di Lullo, Luca; Di Iorio, Biagio; Bellasi, Antonio; Guaraldi, Giovanni	Kidney Disease in HIV Infection	CKD; chronic kidney disease; nephrotoxicity; HIV; antiretroviral therapy
24	2019 [14]	Expósito, Carmen; Pera, Guillem; Rodríguez, Lluís; Arteaga, Ingrid; Martínez, Alba; Alumà, Alba; Doladé, María; Torán, Pere; Caballeria, Llorenç	Prevalence of Early Chronic Kidney Disease and Main Associated Factors in Spanish Population: Populational Study	chronic kidney disease; prevalence; associated factors; albuminuria; obesity; arterial hypertension; type 2 diabetes
25	2019 [22]	Di Iorio, Biagio; Rocchetti, Maria; De Angelis, Maria; Cosola, Carmela; Marzocco, Stefania; Di Micco, Lucia; di Bari, Ighli; Accetturo, Matteo; Vacca, Mirco; Gobbetti, Marco; Di Iorio, Mattia; Bellasi, Antonio; Gesualdo, Loreto	Nutritional Therapy Modulates Intestinal Microbiota and Reduces Serum Levels of Total and Free Indoxyl Sulfate and P-Cresyl Sulfate in Chronic Kidney Disease (Medika Study)	CKD; microbiome; indoxyl sulfate; P-cresyl sulfate; very low protein diet; Mediterranean diet
